# Full-Length Transcriptome Analysis of *Plasmodium falciparum* by Single-Molecule Long-Read Sequencing

**DOI:** 10.3389/fcimb.2021.631545

**Published:** 2021-02-23

**Authors:** Mengquan Yang, Xiaomin Shang, Yiqing Zhou, Changhong Wang, Guiying Wei, Jianxia Tang, Meihua Zhang, Yaobao Liu, Jun Cao, Qingfeng Zhang

**Affiliations:** ^1^Research Center for Translational Medicine, Key Laboratory of Arrhythmias of the Ministry of Education of China, East Hospital, Tongji University School of Medicine, Shanghai, China; ^2^State Key Laboratory of Drug Research, Shanghai Institute of Materia Medica, Chinese Academy of Sciences, Shanghai, China; ^3^CAS Key Laboratory of Synthetic Biology, CAS Center for Excellence in Molecular Plant Sciences, Chinese Academy of Sciences, Shanghai, China; ^4^National Health Commission Key Laboratory of Parasitic Disease Control and Prevention, Jiangsu Provincial Key Laboratory on Parasite and Vector Control Technology, Jiangsu Institute of Parasitic Diseases, Wuxi, China; ^5^Center for Global Health, School of Public Health, Nanjing Medical University, Nanjing, China

**Keywords:** *Plasmodium falciparum*, small protein, long non-coding RNA, alternative splicing, full-length RNA-seq

## Abstract

Malaria, an infectious disease caused by *Plasmodium* parasites, still accounts for amounts of deaths annually in last decades. Despite the significance of *Plasmodium falciparum* as a model organism of malaria parasites, our understanding of gene expression of this parasite remains largely elusive since lots of progress on its genome and transcriptome are based on assembly with short sequencing reads. Herein, we report the new version of transcriptome dataset containing all full-length transcripts over the whole asexual blood stages by adopting a full-length sequencing approach with optimized experimental conditions of cDNA library preparation. We have identified a total of 393 alternative splicing (AS) events, 3,623 long non-coding RNAs (lncRNAs), 1,555 alternative polyadenylation (APA) events, 57 transcription factors (TF), 1,721 fusion transcripts in *P. falciparum*. Furthermore, the shotgun proteome was performed to validate the full-length transcriptome of *P. falciparum*. More importantly, integration of full-length transcriptomic and proteomic data identified 160 novel small proteins in lncRNA regions. Collectively, this full-length transcriptome dataset with high quality and accuracy and the shotgun proteome analyses shed light on the complex gene expression in malaria parasites and provide a valuable resource for related functional and mechanistic researches on *P. falciparum* genes.

## Introduction

Malaria is still a major threat to public health globally caused by *Plasmodium* genus with the occurrence of artemisinin resistance (van der Pluijm et al., 2020). *Plasmodium*, especially *P. falciparum*, is one of the deadliest pathogens that causes malaria in humans which is a disease transmitted by *Anopheles* mosquitoes. Therefore, potential mechanistic regulation pathways should be researched urgently (White et al., 2014). *P. falciparum* as a research model specie of malaria disease was widely studied in a long period, however, the infection and resistance mechanism are still unclear entirely, which attributes to the great adaptation ability of *P. falciparum* to evade host immunity and develop drug resistance. Further understanding of *P. falciparum* will give us clues on discovering the new therapy to cure malaria.

In the last two decades, second-generation sequencing approaches were widely used in genome and transcriptome sequencing which assisted us furtherly understanding the molecular mechanism and function of unknown genes. However, sequences obtained by second-generation short reads assembly always lead to errors so that we could not obtain the full-length transcripts directly and characterize the gene structure accurately, such as the alternative splicing events. RNA-seq as a routine approach was widely used in research of gene discovery and biological functions. Recently, the full-length RNA-seq platform showed advantages in biological research, especially in gene structure identification, gradually taking the place of short-read RNA sequencing in transcriptome profiling.

The understanding of the infection was hindered by high variable and repetitive sequences in the *P. falciparum* genome in previous genetic studies by using short-read sequencing platform. As known, gene structural variations (alternative splicing, alternative polyadenylation, lncRNA and gene fusion, etc.) in transcriptional process resulted in transcriptome complexity which affects the gene function and gene expression regulation (Ma et al., 2018). Gene structural variations were proved to drive genomic diversity in *P. falciparum* (Miles et al., 2016). Besides, mRNA polyadenylation is a universal phenomenon in the transcriptional process in eukaryotes. For the understanding of the mRNA polyadenylation, the high throughout sequencing study of *Sarcocystis neurona*, a unicellular parasite, was performed and it indicated that alternative polyadenylation (APA) is a common phenomenon in unicellular parasites that has the potential to impact growth and development.

Recently, it was revealed that non-coding RNAs play an important role in biological processes and gene function regulation in Apicomplexan parasites by experimental and sequencing technologies (Li et al., 2020). As for *Plasmodium* species, various strategies were carried out for non-coding RNA characterization (Mourier et al., 2008; Raabe et al., 2010; Liao et al., 2014; Siegel et al., 2014; Broadbent et al., 2015; Chappell et al., 2020). Gene fusion is a common phenomenon which was overlooked for a long time. This phenomenon was confirmed with the development of long reads sequencing approach (Rhoads and Au, 2015). For decades, open reading frames (ORF, > 100 codons) were considered as coding sequences which can be translated into proteins (Cabrera-Quio et al., 2016; Yin et al., 2019). However, amounts of small open reading frames (<100 codons) were also produced in the transcriptional stages which were dismissed by current bioinformatic algorithms and always considered meaningless because known functional proteins longer than 100 amino acids (Frith et al., 2006; Ladoukakis et al., 2011). The first research on sORFs were carried out on baker’s yeast, revealing 299 sORFs not annotated before (Kastenmayer et al., 2006). Later, sORFs with high potential of encoding microproteins were found in kinds of organisms like bacteria, insects, plants, and human (Cabrera-Quio et al., 2016; Hsu and Benfey, 2018; Fesenko et al., 2019; Miravet-Verde et al., 2019; Ruiz-Orera and Alba, 2019; Segonzac and Monaghan, 2019; Orr et al., 2020; Patraquim et al., 2020). Among them, it was reported that one small peptide that regulates metabolism and reduces obesity (Lee et al., 2015). Based on these research results, we predicted that sORFs or small proteins probably play an important role in growth development and infection processes in *P. falciparum* which were ignored for a long period. To further understand the functional genes and exploit the infection mechanism of *P. falciparum* for drug discovery and new therapy development, it is necessary to obtain the full-length transcriptional isoforms of its genes and characterize the gene structures.

Defining all the transcripts expressing in the whole asexual blood stages of *P. falciparum* with full length would avoid the assembly errors and assist significantly in understanding the malaria infection process. Herein, we performed the full-length transcriptome sequencing to characterize the full-length transcripts and uncovered alternative splicing, long non-coding RNA, alternative polyadenylation (APA) sites. Besides, combining the full-length transcriptome and the shotgun proteome approaches were used to validate the small proteins coded by CDS in long non-coding RNA. In this process, a modified cDNA library construction procedure for TA-rich species and mixtures of samples at six time points in the whole asexual blood stages were applied into *P. falciparum* resequencing. This work broadens our knowledge far beyond the existing resources in terms of accuracy (full-length sequencing without assembly). Collectively, we not only systematically characterize the complexity of the transcriptome and proteome but also provide a valuable resource for investigating the infection mechanisms of *Plasmodium* parasites.

## Materials and Methods

### Parasites Culture and Collection

*P. falciparum* 3D7 strains were used in this study. *P. falciparum* parasites were grown in 5% O^+^ human erythrocytes in RPMI1640/25 mM Hepes supplemented with 0.5% Albumax I, and were cultured *in vitro* at 37°C under a gaseous mixture of 5% O_2_, 5% CO_2_, and 90% N_2_. Parasites were repeatedly synchronized with 5% sorbitol treatment in ring stage during two consecutive lifecycles when grown at 3–5% parasitemia and then maintained culturing in 175 cm^2^ flasks. After reinvasion, the parasites were started to collect mainly in ring stage when grown at 5–8% parasitemia and then collected by every 8 h. We collected six time-point (8, 16, 24, 32, 40, and 48 hpi) samples with a time window of ~8 h which may cover all stages of parasites in an intraerythrocytic lifecycle. Two biological replicates of pelleted parasites were stored in TRIzol reagent (Invitrogen) at −80°C prior to RNA isolation. Meanwhile, aliquots of the mixed samples were also used for total protein extraction. The proteome extracted from *P. falciparum* mixtures of two biological replicates were used for proteome profiling.

### RNA Preparation and cDNA Library Construction

Total RNA from six time-points (8, 16, 24, 32, 40, and 48 hpi) were prepared by treated in TRIzol reagent and processing according to the manufacturer’s instruction of Zymo RNA Extract kit. To remove genomic DNA, each sample was treated with RNase-free DNase I digestion for 15 min at room temperature and eluted with 50 ul RNase-free water (Invitrogen). Each total RNA was quantified and assessed using an Agilent Bioanalyzer 2100, and then the six RNA samples were pooled into one sample with equal amounts for further library construction. The full-length cDNA was synthesized and library amplified by using the SMARTer PCR cDNA Synthesis Kit (Clontech, CA, USA). After purification, the BluePippin Size Selection System (Sage Science, MA, USA) was used for selection and the cDNA library was constructed by using SMARTbell Template Prep kit (Clontech, CA, USA). The cDNA library was sequenced on PacBio Sequel platform. To obtain the sequencing data with better quality, the optimized method for TA-rich species was applied: an AT-rich optimized KAPA protocol using KAPA HiFi HotStart ready mix (KAPA Biosystems, KM2602) with the following PCR program: 95°C for 5 min; 14 cycles of 95°C for 10 s, 65°C for 1 min; 65°C for 5 min to reduce the bias in the process of cDNA library construction, which improved the coverage of RNA-seq notably.

### PacBio Sequencing Processing and Transcriptome Analysis Pipeline

Sequencing data were processed using the SMRTlink 5.0 software. Circular consensus sequence (CCS) were generated from subread BAM files, parameters: min_length 200, max_drop_fraction 0.8, no_polish TRUE, min_passes 1, min_zscore -9999, min_passes 1. Min_predicted_accuracy 0.8, max_length 18000. CCS.BAM files were output, which were then classified into full length (as defined by reads both with 5’ primer, 3’ primer, and a polyA tail) and non-full length reads using pbclassify.py script, ignore polyA false, minSeq Length 200. Non-full length and full-length fasta files produced were then fed into the cluster step, which dose isoform-level clustering (ICE), followed by final Arrow polishing, hq_quiver_min_accuracy 0.99, bin_by_primer false, bin_size_kb 1, qv_trim_5p 100, qv_trim_3p 30. The misread of nucleobases are much higher in PacBio sequencing reads than in shorter Illumina sequencing reads and can lead to incorrectly detected gene structures. The sequencing errors in the consensus reads were corrected using the Illumina RNA-seq data with the software LoRDEC (Salmela and Rivals, 2014). The corrected consensus reads were then aligned to reference genome using GMAP with parameters: –no-chimeras –cross-species –expand-offsets 1 –B 5 –K 50000 –f samse –n 1 against reference genome (Wu and Watanabe, 2005). The GMAP output bam format file and gff/gtf format genome annotation file were used for gene and transcript determination. All transcripts were mapped on reference genome of *P. falciparum* and unmapped transcripts without overlapping were considered as novel genes. Novel gene transcripts function were annotated based on the following databases: NR, NT, Pfam, KOG/COG, Swiss-Prot, KO, and GO database.

### Characterization of Alternative Splicing Events

SUPPA was used to calculate expression weight (Psi) of alternative splice based on transcript TPM values (Alamancos et al., 2015). Differential alternative splice of two conditions was performed using significance test of Psi. The dpsi value was adjusted using the Mann-Whitney U test method. The absolute dpsi value of 0.1 and p-value of 0.05 were set as the threshold for significantly differential alternative splice. Alternative splicing events were classified into SE (skipped exon), MX (mutually exclusive exon), A5 (alternative 5’ splice site), A3 (alternative 3’ splice site), RI (retained intron), AF (alternative first exon), AL (alternative last exon).

### Alternative Polyadenylation Sites Detection and Transcription Factor Identification

Alternative polyadenylation (APA) sites detection was performed using TAPIS pipeline (Abdel-Ghany et al., 2016). Transcription factors (TFs) were identified and assigned into different families by HMMER 3.0 (Eddy, 2009).

### Gene Fusion Characterization

Fusion transcripts were determined as transcripts mapping to two or more long-distance range genes (Weirather et al., 2015). All the consensus sequences were used for fusion transcripts identification and the criteria used in the process was as follows: (a) a full-length transcript must be mapped to two or more loci on the *P. falciparum* genome; (b) minimum coverage for each locus is 10% of the full-length transcripts; (c) > = 99% total coverage of the full-length transcript was mapped on the *P. falciparum* genome; (d) the distance between each locus mapped on the *P. falciparum* genome is more than 100 kb.

### LncRNA Identification From PacBio Sequences

We used CNCI (Coding-Non-Coding-Index), CPC (Coding Potential Calculator), Pfam-scan, and PLEK four tools to predict the coding potential of transcripts. We use CNCI with default parameters (Sun et al., 2013). We used the NCBI eukaryotes’ protein database and set the e-value “1e-10” in CPC analysis (Kong et al., 2007). Pfam searches used default parameters of –E 0.001 –domE 0.001 (Finn et al., 2016). PLEK used parameters of –minlength 200 (Li et al., 2014). Transcripts predicted with coding potential by either/all of the three tools above were filtered out, and those without coding potential were our candidate set of lncRNAs.

### Global Proteomic Profiling of *Plasmodium falciparum*

*P. falciparum* cultures in different growth stages (8, 16, 24, 32, 40, and 48 hpi) were collected. The proteomics experiment was performed in biological duplicates. Then the cultures were washed with PBS, harvested and lysed with SDT lysis buffer (100 mM Tris-HCl pH 7.6, 4% SDS, 0.1 M DTT) at 95°C for 3 min. The lysates were centrifuged at 14,000 g for 15 min and the supernatants were collected. Each of 300 μg of protein was alkylated with 55 mM of iodoacetamide and subjected to in-solution tryptic digestion utilizing the FASP (filter aided sample preparation) protocol (Wisniewski et al., 2009). The digested peptides were combined and fractionated by high-pH reversed-phase chromatography on a 1-mm Xbridge column (Waters), and eight fractions were collected. Each fraction was evaporated to dryness on a SpeedVac and dried peptides were resuspended in 15 µl of ddH2O containing 0.1% formic acid with sonication for subsequent MS analysis. A volume of 1 μl of each sample was desalted by loading on a Thermo C18 PepMap100 precolumn (300 µm × 5 mm) and eluted on a Thermo Acclaim PepMap RSLC analytical column (75 µm × 15 cm). Mobile phase A (0.1% formic acid in H2O) and mobile phase B (0.1% formic acid in acetonitrile) were used to establish the 120 min gradient comprised of 85 min of 4−30% B, 15 min of 30−50% B, and 5 min of 90% B, followed by re-equilibrating at 4% B for 15 min. The flow rate was 0.3 μl/min. Peptides were then analyzed on Thermo Orbitrap Fusion Lumos proteomic mass spectrometer (Thermo Scientific) in a data-dependent manner, with automatic switching between MS and MS/MS scans using a cycle time 3 s. MS spectra were acquired at a resolution of 120,000 with AGC target value of 4 × 105 ions or a maximum integration time of 50 ms. The scan range was limited from 375 to 1,500 m/z. Peptide fragmentation was performed *via* high energy collision dissociation (HCD) with the energy set at 38 NCE. The MS/MS spectra were acquired at a resolution of 50,000 with AGC target value of 1 × 10^5^ ions or a maximum integration time of 105 ms. The fixed first m/z was 120, and the isolation window was 0.7 m/z.

Protein identification and quantification were performed using Proteome Discoverer 2.1 software (Thermo Scientific). Peptide sequences (and hence protein identity) were searched against the protein database constructed by using the PacBio sequencing and the database of small protein (<100 amino acids) from identified lncRNA with the acquired fragmentation pattern by SEQUEST HT algorithm. The precursor mass tolerance was set to 10 ppm and fragment ion mass tolerance to 0.02 Da. One missed cleavage site of trypsin was allowed. Oxidation (M) was used as variable modifications. All spectra were searched against protein database using a target false discovery rate (FDR) of 1%. The proteins identified in both channels were additionally filtered by at least two spectral counts and one unique peptide in each experimental replicate. Protein ratios were calculated as the median of peptide with S/N ratio higher than 10 of a protein.

### Proteome Analysis and Potential Small Proteins Validation

For global profiling of *P. falciparum* and validating the transcripts identified by full-length transcriptome sequencing, the transcripts with open reading frames (ORF, >300 bp) were translated to construct the protein database. Firstly, all the proteins characterized by liquid chromatography-mass spectrometry were discovered by searching against the protein datasets. And then, these sequences were annotated by KEGG database (Kyoto encyclopedia of genes and genomes) by using BLAST and classified into different KEGG pathway.

To identify small ORFs with high coding potential, the long non-coding RNA (lncRNA) sequences were used to predict the small proteins by selecting the sequences with small open reading frames (ORF, <300 bp). It is considered that these small open reading frames were likely to be translated into small proteins. And then, all of the small ORFs were translated as the small protein (<100 amino acids) database. For small protein detection and validation, these small proteins were validated by using the peptides searching against the small protein database.

### Gene Structure Visualization

The structures of chromosomes, alternative splicing sites, alternative polyadenylation, novel transcripts distribution, novel genes distribution, lncRNA density, and gene fusion were visualized by Circos (Krzywinski et al., 2009).

## Results

### Sample Preparation and PacBio Sequencing

**Figure 1:**
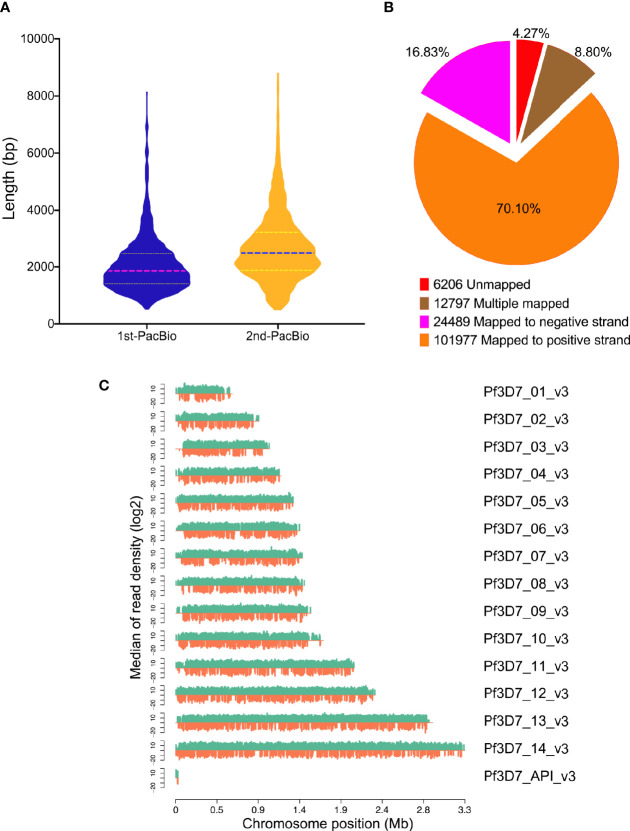
Mapping results of PacBio sequencing. **(A)** Reads length distribution of PacBio sequencing by two different library construction methods; **(B)** GMAP mapping rates; **(C)** Reads distribution on chromosomes of *P. falciparum*. X-axis indicates the chromosome position, y-axis indicates the median of reads density.

To further understand the mechanism and discover the novel genes in *P. falciparum*, the study was designed to perform the full-length transcriptome sequencing by collecting the samples at six time points over the whole asexual blood stages (6, 12, 18, 24, 36, and 48 h). The time window of each sample is 6 h. Two approaches were applied into cDNA library construction and the transcriptome sequencing was performed on PacBio Sequel. For the conventional library construction (1st-PacBio), a total of 18.69 Gb clean data were generated by the mixed sample. The genome of *P. falciparum* is extremely TA-rich, so we carried out the sequencing again by using optimized method (2nd-PacBio) for cDNA library construction and a total of 24.56 Gb clean data was generated. The length distribution of the PacBio sequencing reads from the PacBio sequencing data by using the different cDNA library construction approaches were compared (**Figure 1A**), and it indicated that the optimized library construction method improved the transcriptome data quality a lot. Thus, the sequencing data obtained from the optimized cDNA library approach was used for following analysis.

### PacBio Sequencing and GAMP Mapping

After filtering using the subreads, 7,309,966 subreads were obtained. Next, the Circular consensus sequence (CCS) was generated using the SMRTlink software and the CCS was classified into full-length and non-full length reads according to the 5′ and 3′ adapters and the poly(A) tails. A total of 376,592 circular consensus sequences (CCS) reads were generated and 299,462 (79.5%) sequences were considered as full-length transcripts. A total of 145,469 polished CCS reads with average size 2,387 bp ranging from 155 to 14,521 bp were obtained. The statistics information of transcriptome in detail were summarized in **Supplementary Table 1**.

A total of 145,469 polished CCS reads were searched against *P. falciparum* genome and 139,263 (95.73%) transcripts were mapped on reference genome by GMAP (**Figure 1B**) and the results were summarized in **Supplementary Table 2**. In addition, the reads density showed that all the reads were distributed on the chromosomes homogeneously (**Figure 1C**).

### Alternative Splicing Events Analysis

**Figure 2:**
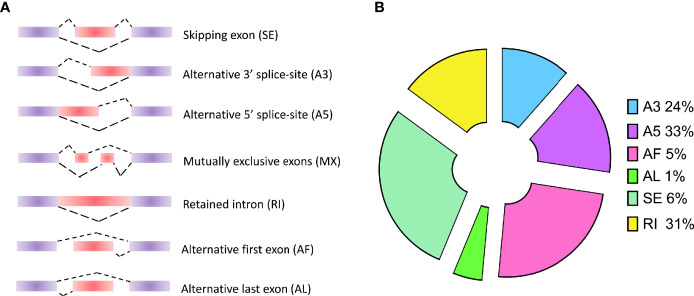
Types of alternative splicing (AS) events and the classification of AS events in *P. falciparum*. **(A)** Types of AS events: SE (skipping exon), MX (mutually exclusive exons), A5 (alternative 5’ splice-site), A3 (alternative 3’ splice-site), RI (retained intron), AF (alternative first exon), and AL (alternative last exon). **(B)** The distribution of AS events in *P. falciparum*.

As known, alternative splicing plays an important role in the process of differentiation and growth in multicellular organisms. For the unicellular protozoa, the study in *P. berghei* indicates that alternative splicing is a stage-specific phenomenon regulating the cellular differentiation into variable cell types (Yeoh et al., 2019). Although alternative splicing events have been studied in *P. falciparum*, only short-read sequencing technologies were applied into detecting the AS events by short reads assembly. In this study, long-read sequencing platform was employed to improve the detection accuracy. The results provide an accurate AS events of all the genes by aligning all the transcripts on the reference genome. Usually, AS events will be classified into seven categories (**Figure 2A**): SE (skipping exon), MX (mutually exclusive exons), A5 (alternative 5’ splice-site), A3 (alternative 3’ splice-site), RI (retained intron), AF (alternative first exon), and AL (alternative last exon). In our study, a total of 393 AS events were detected in *P. falciparum* (**Supplementary Table 3**). Based on the classification and statistics, the AS events were divided into different types. Among these types, A3 (24%), A5 (33%), and RI (31%) were dominant in these AS events (**Figure 2B**). Among them, Apetala 2 (AP2) encode a set of transcription factors in Apicomplexa including *P. falciparum* and the AS events of these genes were visualized: PF3D7_0730300 (A5), PF3D7_0420300 (RI), PF3D7_0613800 (A5), and PF3D7_1239200 (AF) (**Supplementary Figure 3**).

### Long Non-coding RNA Analysis

**Figure 3:**
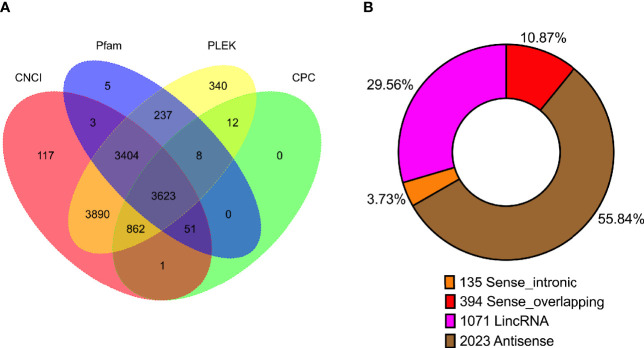
Statistics of long non-coding RNA (lncRNA) in *P. falciparum*. **(A)** Venn diagram showing the number of lncRNAs predicted using four algorithms (CNCI, Pfam, PLEK, and CPC). **(B)** The number of lncRNA classified into sense intronic, sense overlapping, lncRNA, and antisense.

In previous study, unlike those protein-coding RNAs, non-coding RNAs especially lncRNAs are still not well investigated. Though lots of lncRNAs were characterized by the second-generation sequencing, amounts of lncRNAs are still not fully characterized as well as un-correctly because of sequencing shortness. We compared the length distribution (**Supplementary Figure 4A**) and the exon number (**Supplementary Figure 4B**) of mRNA and lncRNA in PacBio sequencing data. To identify lncRNAs in the full-length sequencing transcriptome and obtain the high-confidence lncRNA dataset, four algorithms including Coding-Noncoding Index (CNCI), Pfam-scan (Pfam), the predictor of long non-coding RNAs and messenger RNAs based on an improved k-mer scheme (PLEK), and Coding Potential Calculator (CPC) were employed to characterize the lncRNAs. Among 12,553 potential lncRNAs predicted by four algorithms, the intersection of 3,623 lncRNAs (**Figure 3A**, **Supplementary Table 4**) were identified and were divided into four categories: Antisense (2,023, 55.84%), lncRNA (1,071, 29.56%), sense-overlapping (394, 10.87%), and sense-intronic (135, 3.73%) (**Figure 3B**, **Supplementary Table 4**).

### Alternative Polyadenylation and Transcription Factors Identification

**Figure 4:**
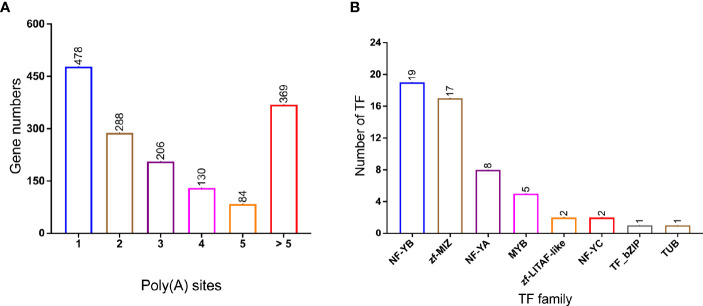
Alternative polyadenylation (APA) and transcription Factor (TF) in PacBio transcriptome. **(A)** Distribution of poly(A) with different poly(A) sites’ number; **(B)** TF family classification of genes. Number of TF genes in each families, NF-YB and zf-MIZ proteins make up a large proportion.

Differential alternative polyadenylation (APA) of mRNAs has been proved to play an important regulatory role in different species (Shen et al., 2011; Elkon et al., 2013). In this study, 1,555 APA events were detected and annotated in **Supplementary Table 5**. Genes with different number of Poly(A) sites were visualized in **Figure 4A** and 478 genes contain 1 poly(A) sites were dominant in all APA events. In addition, 369 genes contain more than five poly(A) sites. A total of 55 variant transcripts of TFs were identified and assigned into different families (**Figure 4B**, **Supplementary Table 6**): NF-YB (19 members), zf-MIZ (17 members), NF-YA (8 members), and MYB (5 members) were the most abundant in *P. falciparum*.

### Full Scan of Proteome

**Figure 5:**
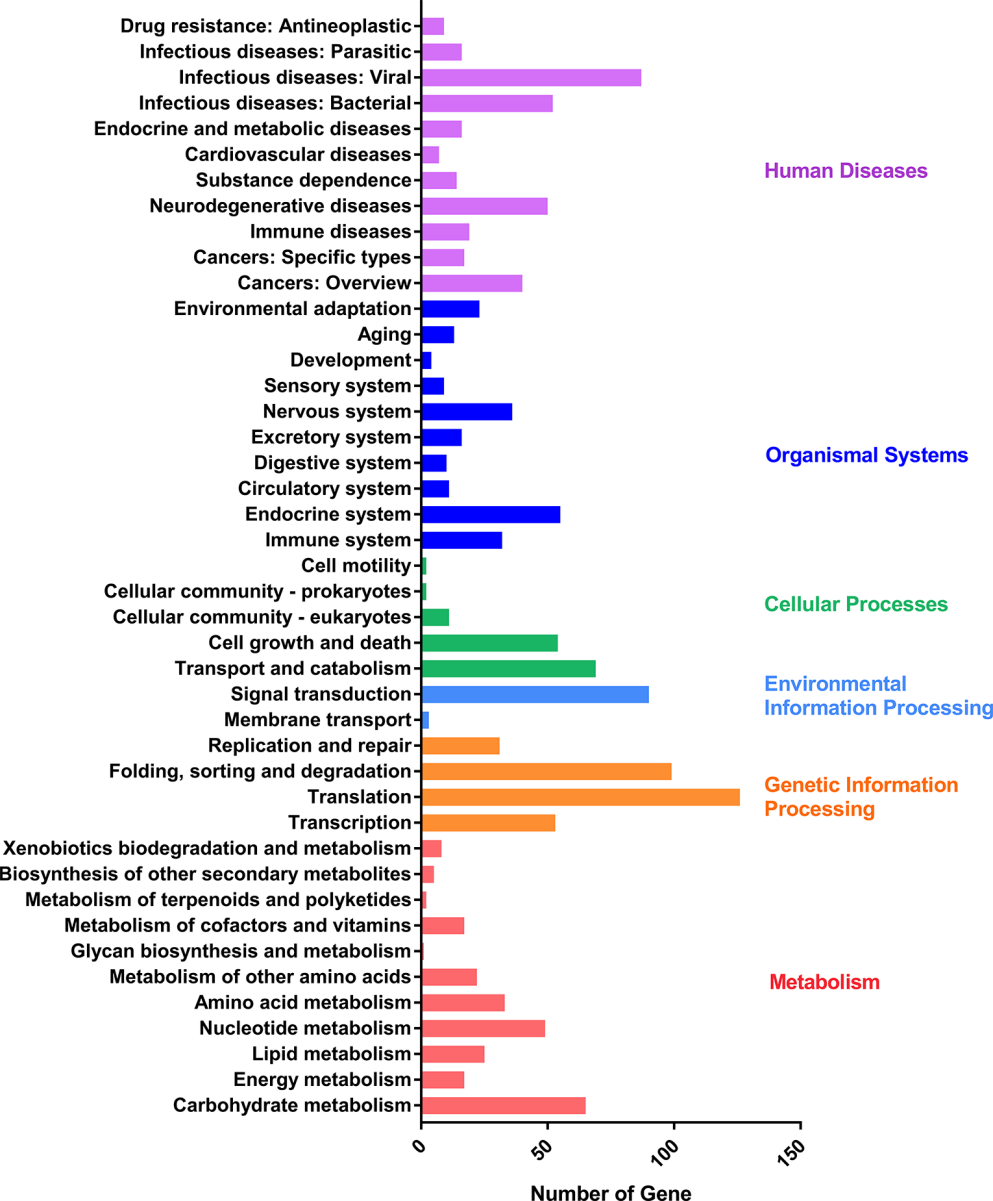
KEGG pathway assignment of proteins characterized by liquid chromatography-mass spectrometry. The bottom x-axis indicates the number of proteins. The left y-axis indicates the categories in detail, the right y-axis indicates the main clustered group of the specific categories.

To validate the full-length sequencing data, the full scan genome was performed with the mixed samples containing the whole asexual blood stages. Proteins in the mixed sample were characterized by Lumos and the spectrums were searched against the protein database constructed by full length sequencing data of *P. falciparum*. A total of 1,535 proteins with high confidence were characterized and list in **Supplementary Table 7**. And then, KEGG analysis was performed by using the proteome data which were classified into six main categories: human diseases, organismal systems, cellular processes, environmental information processing, genetic information processing, and metabolism **(****Figure 5****)**.

### Small Proteins in Long Non-coding RNA

Recently, small proteins (<100 amino acids) were characterized in different species and proved to be functional (Kastenmayer et al., 2006; Fesenko et al., 2019; Sberro et al., 2019; Martinez et al., 2020). *P. falciparum* as a very important species related to human health, however, the small proteins in *P. falciparum* are still unexplored. Thus, small proteins were analyzed in this study and 160 small proteins (<100 aa) were validated by searching against the small protein database (**Supplementary Table 8**).

### Fusion Transcript Identification

**Figure 6:**
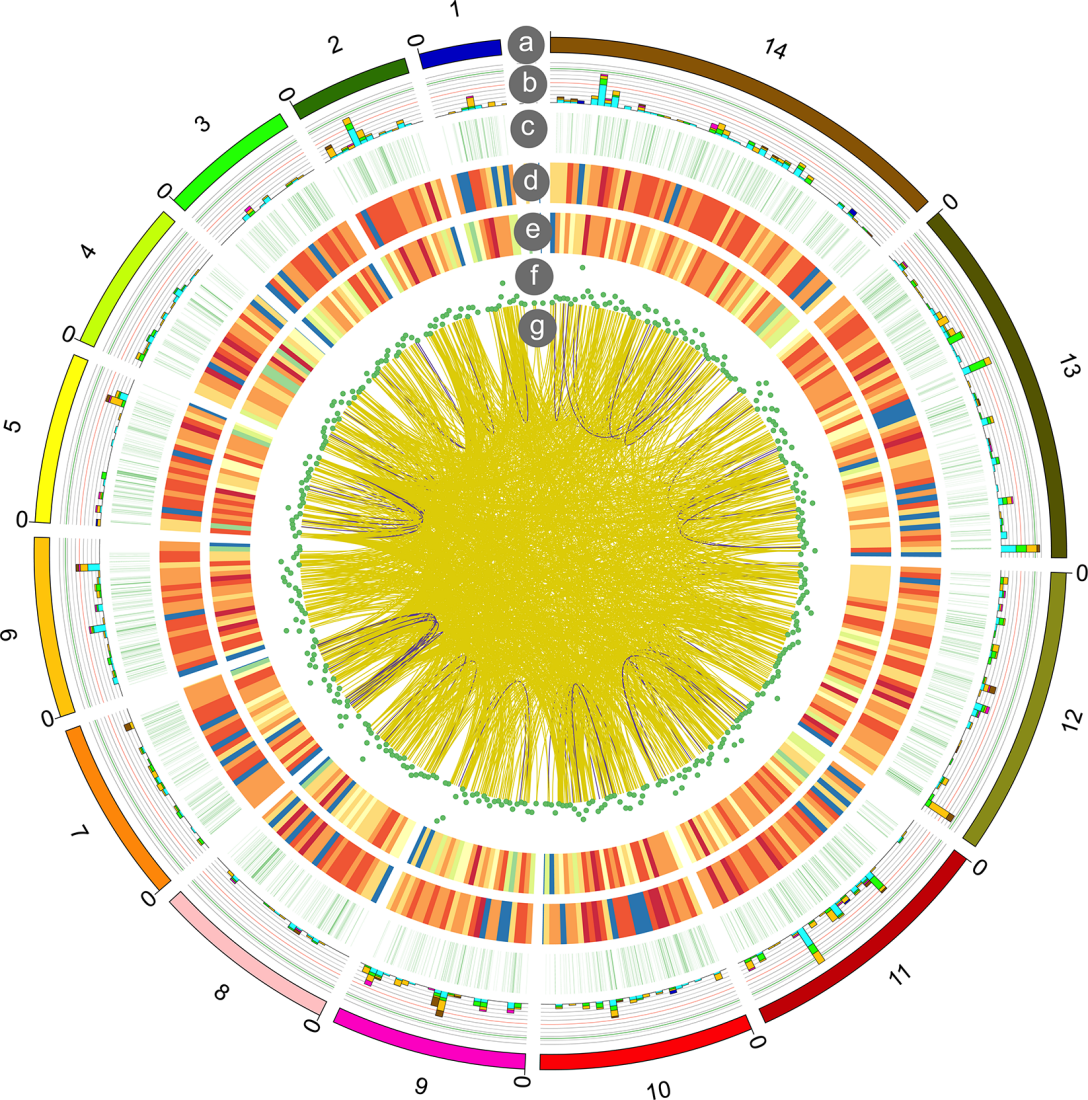
Circos visualization of *P. falciparum* PacBio sequencing results. From outside to inside, the circles represent chromosomes (a); alternative splicing (AS) sites (b); alternative polyadenylation (APA) (c); novel transcripts distribution (d); novel genes distribution (e); long non-coding RNA (lncRNA) density (f); gene fusion distribution (g): intra-chromosome (purple), inter-chromosome (yellow).

Fusion transcripts, usually caused by chromosomal rearrangements, have been proved to play an important role in oncogenesis (Friedrich and Sonnhammer, 2020). In our study, a total of 1,721 fusion transcripts were identified by long-read sequencing. The fusion transcripts were distributed in different chromosomes and shown in **Figure 6**. Among them, 84 and 1,636 fusion transcripts were located in the intra- and inter-chromosomic region (**Supplementary Table 9**).

## Discussion

Currently, the mechanisms of *Plasmodium* parasite invasion and hijacking of host cells, transmission, and immune evasion remain largely elusive. These processes are regulated precisely by a complex dynamic system. Fortunately, a full genome sequence of *P. falciparum* has been sequenced and partial genes have been annotated functionally, which may reveal some underlying mechanisms for physiological activities of malaria parasites (Gardner et al., 2002). However, in most cases, the routine approach of sequencing is not capable of generating reads corresponding to entire transcripts because of the short reads which will result in mistakes during the assembly (van Dijk et al., 2014). Now, the single-molecule, real-time (SMRT) sequencing technique producing kilobase-sized reads has been developed (Eid et al., 2009), which help us obtain the entire transcripts without assembly easily (Sharon et al., 2013). As for the malaria parasites, though the genome annotation of *P. falciparum* has been updated frequently based on more and more microarray or RNA-seq data since 2002 (Gardner et al., 2002), there are still many annotation mistakes. This interferes those researches on gene function or underlying mechanism. Here, the updated full-length transcriptome provides a valuable resource for further studies on gene regulation and protein functions in the human malaria parasites.

Long non-coding RNA (lncRNA) were proved to play a role in transcriptional regulation in eukaryotic organisms including *P. falciparum* (Broadbent et al., 2015). For instance, the antisense lncRNAs produced by the intronic promoters of *var* genes were involved in the transcriptional activation of these virulence genes (Jing et al., 2018). To date, the non-coding transcriptome and their biological functions remain largely unknown. In our study, 3,623 full-length lncRNA were characterized by using four algorithms (CNCI, Pfam, PLEK, and CPC) and were divided into four categories including Antisense, lncRNA, sense-overlapping, and sense-intronic transcripts. This data will facilitate the functional studies of lncRNAs in malaria parasites. More importantly, lncRNA-derived small proteins have been validated by integration of full-length transcriptomic and proteomic data in this study. Until then, small proteins are often ignored in transcriptome. Here, the proteome profiling was applied successfully to characterize 1,535 proteins with high confidence. Besides, 160 small proteins were validated by liquid chromatography-mass spectrometry corresponding to lncRNA regions of *P. falciparum*. These results not only provide an updated proteome database within the whole asexual blood stages, but also assist the small protein discovery and functional investigation in *P. falciparum*.

Finally, to our knowledge, this is the first report of the PacBio full-length transcriptome in *Plasmodium*, which compensates the deficiency of the conventional sequencing methods using Illumina-generated short reads in terms of gene expression. Many of the predicted transcripts that did not correspond to the annotated transcripts may potentially regulate *P. falciparum* development and reveal a complex transcriptional landscape in the asexual blood stage. However, the underlying molecular mechanism of alternative splicing, long non-coding RNAs, alternative polyadenylation, and fusion transcripts still requires more sufficient research to investigate in the future, especially how these novel proteins identified by multi-omics analysis are involved in parasites growth and development process. We believe this work provides a novel and valuable genetic resource for functional and mechanistic studies of genes of interest in *P. falciparum*.

## Conclusion

In our study, full-length RNA sequencing was used to reconstruct the transcriptome of *P. falciparum*. An improved transcriptomic dataset covering the whole asexual blood stages without short-read assembly was obtained. This is the first time to reveal the full-length transcriptome in *P. falciparum*. Among 145,469 transcripts, 139,263 (95.73%) of them were mapped on reference genome, and 393 alternative splicing (AS) events, 3,623 long non-coding RNAs (lncRNA), 1,555 alternative polyadenylation (APA) events, 57 transcription factors (TF), 1,721 fusion transcripts were identified, respectively. In addition, 1,535 proteins with high confidence were validated by liquid chromatography-mass spectrometry. Notably, 160 small proteins were identified by liquid chromatography-mass spectrometry searching against the small protein database. This study not only provides an improved full-length transcriptomic dataset with high quality and accuracy, but also contributes to better understanding of structural variations in the transcription process.

## Data Availability Statement

The raw sequence of transcriptomic data reported in this paper have been deposited in the Genome Sequence Archive in the BIG Data Center (http://bigd.big.ac.cn/gsa/s/4dvmackj), Beijing Institute of Genomics (BIG), Chinese Academy of Sciences, under accession number: CRA003525. Proteomic data are available *via* ProteomeXchange with identifier PXD022618.

## Author Contributions

QZ and JC designed the study. MY, XS, YZ, GW, CW, JT, MZ, and YL performed the experiments and analyzed the data. MY and QZ wrote the manuscript with contributions from the other authors. All authors contributed to the article and approved the submitted version.

## Funding

This work was supported by the National Key R&D Program of China Grant (2018YFA0507300) and National Natural Science Foundation of China (NSFC) (81630063, 81971959, and 31671353) to QZ, National Key R&D Program of China Grant (2020YFC1200105), National Natural Science Foundation of China (NSFC) (81971967), and Jiangsu Provincial Project of Invigorating Health Care Through Science, Technology and Education and the Jiangsu Provincial Commission of Health to JC, National Natural Science Foundation of China (NSFC) (22077011) to YZ.

## Conflict of Interest

The authors declare that the research was conducted in the absence of any commercial or financial relationships that could be construed as a potential conflict of interest.

## Supplementary Material

Supplementary Figure 1 Saturation curve of alignment by GMAP. X-axis indicates the number of full-length reads, y-axis indicates the number of genes.

Supplementary Figure 2 Number of mapped reads in chromosomes. X-axis indicates the chromosome length, y-axis indicates the number of mapped reads.

Supplementary Figure 3 The representative AS events identified in PacBio transcriptome. The AS events of Apetala 2 (AP2) genes: PF3D7_0730300 (A5), PF3D7_0420300 (RI), PF3D7_0613800 (A5), and PF3D7_1239200 (AF).

Supplementary Figure 4 Comparison of the lncRNA and mRNA. **(A)** Length distribution of lncRNA and mRNA; **(B)** Distribution of exons in lncRNA and mRNA.

## Abbreviations

AS, alternative splicing; APA, alternative polyadenylation; LncRNA, long non-coding RNA; TF, transcription factor; NR, NCBI non-redundant protein sequences; NT, NCBI non-redundant nucleotide sequences; Pfam, protein family; KOG/COG, Clusters of Orthologous Groups of proteins; Swiss-Prot, SWISS-PROT Protein Sequence Data Bank; KO, KEGG Ortholog database; GO, Gene Ontology; NCBI, National Center for Biotechnology Information; iProx, Integrated Proteome Resources.
